# Repurposing Auranofin as a Lead Candidate for Treatment of Lymphatic Filariasis and Onchocerciasis

**DOI:** 10.1371/journal.pntd.0003534

**Published:** 2015-02-20

**Authors:** Christina A. Bulman, Chelsea M. Bidlow, Sara Lustigman, Fidelis Cho-Ngwa, David Williams, Alberto A. Rascón, Jr, Nancy Tricoche, Moses Samje, Aaron Bell, Brian Suzuki, K. C. Lim, Nonglak Supakorndej, Prasit Supakorndej, Alan R. Wolfe, Giselle M. Knudsen, Steven Chen, Chris Wilson, Kean-Hooi Ang, Michelle Arkin, Jiri Gut, Chris Franklin, Chris Marcellino, James H. McKerrow, Anjan Debnath, Judy A. Sakanari

**Affiliations:** 1 Center for Discovery and Innovation in Parasitic Diseases, University of California San Francisco, San Francisco, California, United States of America; 2 Lindsley F. Kimball Research Institute, New York Blood Center, New York, New York, United States of America; 3 Department of Biochemistry and Molecular Biology, University of Buea, Buea, SW Region, Cameroon; 4 Department of Immunology and Microbiology, Rush University Medical Center, Chicago, Illinois, United States of America; 5 Department of Chemistry, San Jose State University, San Jose, California, United States of America; 6 FilariaTech, Athens, Georgia, United States of America; 7 Department of Infectious Diseases, University of Georgia, Athens, Georgia, United States of America; 8 Department of Bioengineering and Therapeutic Sciences, University of California San Francisco, San Francisco, California, United States of America; 9 UCSF Mass Spectrometry Facility, Department of Pharmaceutical Chemistry, University of California San Francisco, San Francisco, California, United States of America; 10 Small Molecule Discovery Center, University of California San Francisco, San Francisco, California, United States of America; 11 Case Western Reserve University School of Medicine, Cleveland, Ohio, United States of America; 12 Skaggs School of Pharmacy and Pharmaceutical Sciences, University of California San Diego, San Diego, California, United States of America; University of Melbourne, AUSTRALIA

## Abstract

Two major human diseases caused by filariid nematodes are onchocerciasis, or river blindness, and lymphatic filariasis, which can lead to elephantiasis. The drugs ivermectin, diethylcarbamazine (DEC), and albendazole are used in control programs for these diseases, but are mainly effective against the microfilarial stage and have minimal or no effect on adult worms. Adult *Onchocerca volvulus* and *Brugia malayi* worms (macrofilariae) can live for up to 15 years, reproducing and allowing the infection to persist in a population. Therefore, to support control or elimination of these two diseases, effective macrofilaricidal drugs are necessary, in addition to current drugs. In an effort to identify macrofilaricidal drugs, we screened an FDA-approved library with adult worms of *Brugia* spp. and *Onchocerca ochengi*, third-stage larvae (L3s) of *Onchocerca volvulus*, and the microfilariae of both *O. ochengi* and *Loa loa*. We found that auranofin, a gold-containing drug used for rheumatoid arthritis, was effective *in vitro* in killing both *Brugia* spp. and *O. ochengi* adult worms and in inhibiting the molting of L3s of *O. volvulus* with IC_50_ values in the low micromolar to nanomolar range. Auranofin had an approximately 43-fold higher IC_50_ against the microfilariae of *L. loa* compared with the IC_50_ for adult female *O. ochengi*, which may be beneficial if used in areas where *Onchocerca* and *Brugia* are co-endemic with *L. loa*, to prevent severe adverse reactions to the drug-induced death of *L. loa* microfilariae. Further testing indicated that auranofin is also effective in reducing *Brugia* adult worm burden in infected gerbils and that auranofin may be targeting the thioredoxin reductase in this nematode.

## Author Summary

Onchocerciasis or river blindness, and lymphatic filariasis, which can lead to disfiguring elephantiasis, are two neglected tropical diseases that affect millions of people, primarily in developing countries. Both diseases are caused by filariid nematodes; onchocerciasis is caused by *Onchocerca volvulus* and lymphatic filariasis is caused by *Brugia malayi*, *B. timori*, and *Wuchereria bancrofti*. Currently, there are no drugs available that are highly efficacious against adult worms; existing drugs mainly kill the first-stage larvae (microfilariae). While these drugs can reduce the transmission of infections in a population, the adult filariids (macrofilariae) can continue to produce microfilariae and perpetuate the cycle of infection. Finding a drug that could kill the adult worms would be an important tool in eliminating onchocerciasis and lymphatic filariasis. To identify potential macrofilaricidal drugs, we developed a high throughput screening method to test FDA-approved drugs on adult *Brugia* spp., which serves as a model for *O. volvulus*. Using this screening method, we identified a drug called auranofin that kills adult *Onchocerca* and adult *Brugia* spp. *in vitro*, inhibits the molting of *O. volvulus* L3s, and reduces the worm burden in an *in vivo* gerbil-*B. pahangi* model system. Auranofin is known to inhibit a critical enzyme called thioredoxin reductase in some parasite species, and subsequent testing of the effects of auranofin on the thioredoxin reductase of *Brugia* indicates that this may be auranofin’s mode of action in this nematode as well.

## Introduction

River blindness and lymphatic filariasis (LF) are two major neglected diseases caused by filariid nematodes that, together, affect an estimated 145 million people worldwide in mostly poor, developing countries [1,2]. River blindness, caused by the filariid nematode *Onchocerca volvulus*, is a chronic, debilitating disease and a major cause of infectious blindness. The adult worms, or macrofilariae, reside in subcutaneous tissues where females release the early larval stage, microfilariae, into the skin. Adult worms can reproduce for up to 10–14 years, releasing millions of microfilariae over an infected individual’s lifetime [3]. Microfilariae migrate throughout the tissues and those that accumulate in the eyes induce an inflammatory response that eventually leads to blindness [4]. LF is caused by several species of filariid nematodes: *Wuchereria bancrofti*, *Brugia malayi* and *B. timori*. The adult worms reside in the lymphatic tissues where females release microfilariae into the circulation. The microfilariae are then ingested by mosquitoes and develop into the infectious larval stage. With LF, the chronic condition is characterized by pain and severe lymphedema often involving the arms, legs, breasts and genitalia, as well as elephantiasis, all of which may lead to social stigma and economic loss to those afflicted [4,5].

Currently, global health programs that aim to eliminate these diseases distribute ivermectin, diethylcarbamazine (DEC), and albendazole through mass drug administration (MDA) to reduce transmission and ideally break the cycle of infection [6]. However, these drugs mainly target the microfilarial stage of the parasite, leaving the adult worms to continue to reproduce. DEC can cause adverse effects in patients infected with *O. volvulus*, so it can only be used to treat LF in areas where onchocerciasis is not endemic [4,6]. There is also an increased risk of serious adverse events, including encephalopathy and death, in those individuals who are treated with ivermectin or DEC and are co-infected with *Loa loa* with high microfilaraemia (greater than 30,000 microfilariae per mL) [7–10]. Recently, the veterinary drug, moxidectin has been investigated as a potential new therapeutic for filarial infection. Awadzi et al (2014) found that moxidectin was an effective microfilaricidal drug in a small-scale study, but it could not be concluded that moxidectin was macrofilaricidal or caused sterility in adult worms [11]. The antibiotic, doxycycline, has been shown to be safe and efficacious in treating both lymphatic filariasis and onchocerciasis, and can sterilize and eventually kill adult worms. However, doxycycline requires long treatment periods of upwards of 4–6 weeks, which is unlikely to be feasible for MDA [4]. These factors, in addition to the difficulty of attaining sufficient coverage through MDA, make discovering effective macrofilaricidal treatments to cure infections a high priority in stopping the transmission of filariasis. An ideal drug candidate is one that has high specificity for *Onchocerca* and *Wuchereria/Brugia* macrofilariae, but has little to no effect on the microfilariae of *L. loa*.

The overall goal of our program is to identify lead candidates for the treatment of river blindness and LF. Previously, we developed an *in vitro* worm assay [12] using *Brugia pahangi* and *B. malayi* as a primary screen to identify compounds that inhibit worm motility. The WormAssay apparatus and computer software (Worminator) enables us to screen compounds against adult *Brugia* in 24-well plates in less than one minute and assess worm killing in an objective manner. Compounds that strongly inhibited adult worm motility in a 3-day assay were then tested against molting *O. volvulus* third-stage larvae (L3) and adult *O. ochengi*. Adult *O. ochengi*, which naturally infect cows and develop in subcutaneous nodules, serve as a model organism for *O. volvulus*, which only infects humans and non-human primates [13–15].

In this study, we screened a library of over 2,000 FDA-approved compounds and found that auranofin was highly effective in inhibiting adult *Brugia* motility. Auranofin is an FDA-approved, gold-containing compound (2,3,4,6-tetra-O-acetyl-1-thio-beta-D-glucopyranosato-S (triethylphosphine) gold) that has been used to treat rheumatoid arthritis for over 25 years [16,17]. Orally dosed auranofin is rapidly metabolized *in vivo* but its active metabolite is not known. It has been suggested that triethylphosphine gold or deacetylated auranofin could be the biologically active metabolites and that some form of the gold from auranofin circulates bound to plasma protein [18–20]. Since gold is known to be necessary for auranofin’s drug activity, studies of its pharmacokinetics employ elemental analysis for gold [19,21–24]. Previous studies have shown that the likely target of auranofin is thioredoxin reductase (TrxR) [25,26], which is a key enzyme involved in reducing oxidative damage in cells. We also found that auranofin is effective in killing adult *Brugia* in an *in vivo* gerbil model and that TrxR is most likely the target of auranofin in *Brugia*.

## Methods

### Drug screening of adult *Brugia* worms *in vitro*

Adult female and male *Brugia* (*B. malayi* and *B. pahangi*) were shipped from TRS Labs Inc., Athens, GA and assayed using methods described by Marcellino *et al*. (2012) [12]. Individual females were placed in each well of a 24-well plate with media (RPMI-1640 with 25 mM HEPES, 2.0 g/L NaHCO_3_, 5% heat inactivated FBS, and 1X Antibiotic/Antimycotic solution). Excess media was removed from plates using a Biomek FxP, leaving 500 μL of media per well. Initial screening of a library of FDA-approved compounds, compiled by the Small Molecule Discovery Center at the University of California San Francisco, was conducted at 10 μM per compound, and 1% DMSO was used as a negative control. All drugs including auranofin (Enzo Life Sciences, Farmingdale, NY) were dissolved in DMSO (Sigma-Aldrich, St. Louis, MO) and 10 mM stock solutions were stored at -20°C. Four worms were used as replicates for each concentration and worm plates were kept in a 37°C, 5% CO_2_ incubator for four days. Auranofin was also tested against male *Brugia* worms under the same conditions after initial screening against female *Brugia* revealed its high level of inhibitory activity.

To determine the effect of a compound on worm motility, individual worm movements were counted as the number of pixels displaced per second by each worm in each well using the Worminator. Each plate of worms was video recorded for approximately 60 seconds, and mean movement units (MMUs) were determined for individual worms. Percent inhibition of motility was calculated by dividing the MMUs of the treated worms by the control average MMUs, subtracting the value from 1.0, flooring the values to zero and multiplying by 100%. Videos were recorded for 4 days, including the first day of the assay (Day 0). IC_50_ determinations were conducted at 10 μM, 3 μM, 1 μM, 0.3 μM, 0.1 μM and 0.03 μM, with 1% DMSO used as a control. IC_50_ assays were repeated at the same concentrations and at six point, three-fold dilutions from 1 μM to 0.003 μM or 3 μM to 0.001 μM to ensure that activity was consistent between assays. Prism 4.0 was used to calculate IC_50_ values using a non-linear regression curve fit. The means of all IC_50_s with R^2^ values greater than or equal to 0.7 are reported.

### Drug screening of adult *Onchocerca ochengi in vitro*

Cows that had grazed in northern Cameroon where *O. ochengi* is highly endemic were brought to abattoirs located in Douala, Cameroon. Subcutaneous nodules containing adult *O. ochengi* worms were identified on the umbilical skin of infected cows. Adult worm masses containing one viable, adult female and zero to several adult males were carefully recovered by dissection of the nodule with a sterile razor blade. The masses were then incubated in 4 mL of complete culture medium (CCM), which was comprised of RPMI-1640 (Sigma-Aldrich), 5% newborn calf serum, 200 units/mL penicillin, 200 μg/mL streptomycin and 2.5 μg/mL amphotericin B (Sigma-Aldrich), in standard 12-well culture plates. Masses were maintained in the medium in a 37°C, 5% CO_2_ incubator overnight during which period most of the smaller and more agile adult males migrated out of the masses while the females remained in the nodules. Worm viability was checked microscopically by observing the movement of adult male worms or emergence of viable microfilariae from the nodular masses. The next day, 2 mL of the CCM was removed and replaced with 60 μM auranofin in 2 mL CCM in each well to generate a final drug concentration of 30 μM. The compound and controls were tested in quadruplicate at each concentration and the experiments were repeated twice on different days. The negative control wells received only 1% DMSO. Cultures were terminated on day 7 post addition of drug. Adult male worm viability was visually scored on day 5 as percent reduction of motility ranging from 100% (complete inhibition of motility), 90% (only head or tail of worm moving or vibrating), 75% (worm very sluggish), 50% (worm sluggish), 25% (little change in motility), to 0% (no observable reduction in motility). Adult female worm viability was assessed on day 7 by the standard MTT/formazan assay in which each nodular mass was placed in a well of a 48-well microtiter plate containing 500 μL of 0.5 mg/mL MTT (Sigma-Aldrich) in incomplete culture medium, and then incubated in the dark at 37°C for 30 minutes. Adult female worm viability was evaluated visually by the extent to which the female worm mass was stained with MTT. Mean percent inhibition of formazan formation was calculated relative to the negative control worm masses after 7 days in culture. Adult worm death positively correlated with inhibition of formazan formation.

To calculate the IC_50_ of auranofin, quadruplicate worm masses were incubated with final concentrations of 30 μM, 10 μM, 3 μM, 1 μM, 0.3 μM, 0.1 μM and 0.03 μM and assays were conducted as described above. Prism 4.0 for Windows was used to calculate IC_50_s.

### Drug screening of *Onchocerca ochengi* and *Loa loa* microfilariae *in vitro*

*O. ochengi* microfilariae were obtained from the umbilical skin of infected cattle and cultured on confluent monkey kidney epithelial cells for drug testing as previously described [27].

*Loa loa* microfilariae were purified from the blood of a heavily infected subject (having approximately 10,000 microfilariae/mL of blood) using Percoll (GE Healthcare, Piscataway, NJ) gradient centrifugation. Venous blood (10 mL) was collected from consenting, infected individuals in an EDTA tube. The blood was layered on a step-wise Percoll gradient (46% and 43% Percoll prepared in CCM) followed by centrifugation at 400 rcf for 20 minutes. The *L. loa* microfilariae were recovered in the 43% layer, washed 3 times in CCM and counted.

Microfilariae (10–15 per well) were cultured in 96-well culture plates in duplicate under the same conditions and drug concentrations as were used for the adult *O. ochengi*, except that 10 μg/mL ivermectin was used as a positive control. Microfilariae viability was visually scored based on motility reduction using the same scale described above for adult male *O. ochengi*. Scores were recorded every 24 hours after the addition of drugs for 5 days using an inverted microscope.

### Drug screening of *Onchocerca volvulus* L3 molting *in vitro*

L3 stage larvae previously collected and cryopreserved in Cameroon were rapidly thawed in a 37°C water bath and washed in incomplete media comprised of a 1:1 ratio of Medium NCTC-109 and IMDM + GlutaMax-I containing 1X glutamine, penicillin, and streptomycin (all from Gibco by Life Technologies, Grand Island, NY). The number of worms was adjusted to about 10 worms per 50 μL in complete medium containing 20% heat inactivated FCS. Worms were distributed into the wells of a 96-well plate containing 50 μL of 1.5 × 10^5^ normal human PBMCs. 100 μL of 2X auranofin (final concentrations of 30 μM, 10 μM, 3 μM, 1 μM and 0.3 μM) were added to each well for a final volume of 200 μL. Each concentration was tested in triplicate. Controls included 0.05% DMSO in complete medium and complete medium only with neither DMSO nor compound added. The 96-well plates were then incubated at 37°C in a 5% CO_2_ incubator for 6 days, then molting was assessed using an inverted microscope. Molting was determined in each well by counting the presence of fourth-stage larvae (L4) and empty casts of the L3. The percent inhibition of molting was calculated based on the number of treated larvae that were able to molt in comparison to the number of control larvae that had successfully molted. Prism 4.0 for Windows was used to calculate IC_50_s.

### Ultrastructure of auranofin-treated adult female *Brugia* and *O. ochengi*

Adult female *B. pahangi* worms were incubated with either 1 μM, 0.3 μM, or 0.1 μM auranofin, 10 μM flubendazole (as a positive control [28]), or 1% DMSO overnight, then cut into 3 segments separating the anterior, middle and posterior sections. The middle sections were further cut into 1 mm sized pieces in fixative (2.5% glutaraldehyde and 2% paraformaldehyde in 0.1 M sodium cacodylate buffer, pH 7.3–7.4) and stored at 4°C. Middle sections were subsequently treated with 1% tannic acid for 1 hour, followed by three buffer washes before post fixation staining with 2% osmium tetroxide for 1 hour. The samples were washed three times in buffer before dehydration in an ethanol series. Worm sections were then infiltrated with propylene oxide, embedded in epon 812 resin and polymerized in a vacuum oven at 60°C overnight. Ultrathin sections were cut using an RMC MTX ultramicrotome with a Diatome diamond knife followed by post staining of the grids with saturated ethanolic uranyl acetate and Reynolds lead citrate. Samples were imaged on a FEI Tecnai 12 spirit TEM operated at 80 kV. A similar procedure was performed on adult female *O. ochengi* worm masses that were cultured for 7 days with 10 μM auranofin before fixation of cut pieces of the adult female mass. Untreated adult female masses cultured for 7 days and fixed by the same procedure served as the control.

### *In vivo* studies of *Brugia pahangi* infected gerbils

Animal studies were performed under IACUC approval #AN085723–02 to test the efficacy of auranofin *in vivo*. Male Mongolian gerbils (*Meriones unguiculatus*, Charles River Laboratories International, Inc., Wilmington, MA) were injected intraperitoneally (IP) with 300 *B. pahangi* L3 (Filariatech, Inc., Athens, GA) and treated 3 months post-infection. Auranofin was dissolved in 100% ethanol at 4 mg/mL and mixed 1:1 with PBS. Vehicle doses consisted of the same mixture of ethanol and PBS but without auranofin. Doses (up to 200 μl) were given to gerbils orally at 5 mg/kg body weight, BID weekdays and SID weekends for a total of 48 doses over 4 weeks.

Two studies (Study 1 and Study 2) were conducted using the same protocols and the same dosing schedule except that in Study 1, two gerbils from the auranofin treatment group and two gerbils from the vehicle group were treated for 14 days and were necropsied 2 hours after their last dose (interim necropsy) to determine plasma gold levels (from auranofin). The remaining gerbils in Study 1 were treated for 28 days and were necropsied 11, 14, or 16 days after the end of dosing. In Study 2, all gerbils were treated for 28 days and were necropsied 16 days after the end of dosing. For both of these *in vivo* studies, worms were collected from the gerbil’s peritoneal cavity, counted, sexed and examined under a dissecting microscope. For each study a two-tailed Student’s T-test assuming equal variance was conducted using Microsoft Excel to determine the statistical significance of the difference in mean worm retrieval between the auranofin treated and vehicle treated groups. Gerbil blood was collected by cardiac puncture and plasma was sent to NMS Labs, Willow Grove, PA to determine plasma gold levels (elemental gold analysis) by graphite furnace atomic absorption spectroscopy.

### Target validation of auranofin-treated worms *in vitro* and *in vivo*

Thioredoxin reductase activity of worm lysates was assayed using female *B. malayi* treated *in vitro* with either 0.3 μM, 0.1 μM, or 0.03 μM auranofin or 1% DMSO. After 5 hours of treatment, worm motility was measured using the Worminator, and then worms (24 in each group) were pooled, washed three times in PBS, and lysed by douncing in a glass homogenizer in assay buffer (Abcam Thioredoxin Reductase Assay kit, ab83463) with 1 mM PMSF. The crude lysates were centrifuged at 10,000 rcf for 15 minutes at 4°C to pellet insoluble material. The total protein concentrations of soluble lysates were measured using the Bradford assay. The soluble lysates were incubated for 20 minutes in assay buffer or assay buffer with a proprietary thioredoxin reductase specific inhibitor before adding a specific substrate, DTNB (5, 5′-dithiobis (2-nitrobenzoic) acid), and measuring activity at 20 second intervals for 40 minutes using the SpectraMax Plus Microplate Reader (Molecular Devices, Sunnyvale, CA) at λ = 412 nm. Lysates were tested in duplicate. TrxR activity was calculated based on the linear amount of TNB produced per minute per mg of total protein and adjusted for background activity from enzymes other than TrxR in the lysates.

Thioredoxin reductase activity was also analyzed in worms that were treated with auranofin or vehicle *in vivo*. Adult male and female worms were transplanted intraperitoneally, and gerbils were treated with auranofin or vehicle for 28 days as was done in the previous *in vivo* studies. Gerbils were necropsied 16 days after the final dose, and lysates were prepared from recovered worms and assayed as above.

### Expression of recombinant *Brugia malayi* thioredoxin reductase

The open reading frame for *B. malayi* TrxR (XM_001898694.1) was synthesized (GenScript) with codons optimized for expression in *Escherichia coli*. The two C-terminal amino acids (selenocysteine (Sec)-Gly), missing in XM_001898694.1, were added along with a bacterial SECIS (selenocysteine insertion sequence) to allow expression of the Sec protein in *E*. *coli* in pET100 (Invitrogen by Life Technologies) [29]. For PCR, the reverse primer was 5’-GGCCGCATAGGTTAACGATTGGTGCAGACCTGCAACCGATTATTAACCTCAGCATCCCGTTGCTTTC-3’ and forward primer was 5’-CACCATGCTGCTGCGTTCCAATGC-3’.

To determine if the *B. malayi* TrxR is a selenoprotein, as are some known thioredoxin reductases, a bioinformatics search was conducted to find a SECIS in the *B. malayi* genome near the thioredoxin reductase gene. For a detailed description, please see supplementary information (S1 Text).

Recombinant 6-His-tagged *B. malayi* TrxR (rBmTrxR) was produced in *E*. *coli* BL21(DE3) in the presence of pSUABC [29] in LB medium supplemented with 20 μg/mL riboflavin under conditions for optimal selenoprotein expression [30]. An overnight starter culture in LB with 50 μg/mL ampicillin and 34 μg/mL chloramphenicol was diluted (1:100) into in LB medium with the same antibiotics. When the culture reached an OD_600_ = 0.8, the medium was supplemented with 5 μM sodium selenite and 100 μg/mL L-cysteine. When the culture OD_600_ = 2, riboflavin (20 μg/mL) was added and protein expression was induced by the addition of isopropyl β-D-1-thiogalactopyranoside (50 μM). At this point the cultures were shifted to 24°C and incubated for 24 hr. Cells were collected by centrifugation, lysed by alternative freeze-thaw cycles, and resuspended in lysis buffer (50 mM potassium phosphate, pH 7.8, 500 mM NaCl, 30 mM imidazole, 1 mg/mL lysozyme, 1 mM phenylmethanesulfonylfluoride) supplemented with 100 μM flavin adenine dinucleotide. The sample was sonicated and cellular debris pelleted at 25,000 x g at 4°C for 25 min. The supernatant was collected and filtered through a 0.45 μm filter before purification by immobilized metal ion affinity chromatography using a His-Trap FF column (GE Healthcare). The column was washed with 10 column volumes binding buffer (50 mM potassium phosphate, pH 7.8, 500 mM NaCl, 30 mM imidazole) and then with 5 column volumes of buffer A (binding buffer with 100 mM imidazole). TrxR protein was eluted in 3 × 1 mL buffer B (binding buffer with 500 mM imidazole).Protein was concentrated (Amicon Ultra-4 10K) and purity was verified by SDS-PAGE and quantified by absorbance at 280 nm (ε = 69.76 mM^-1^ cm^-1^).

rBmTrxR activity was assayed in 0.1 M potassium phosphate (pH 7.2) with 10 mM EDTA and 25 nM rBmTrxR. rBmTxrR was pre-incubated for 20 min with NADPH (100 μM) and auranofin (ICN Pharmaceuticals, now Valeant Pharmaceuticals, Bridgewater, NJ) or aurothioglucose (USP Reference Standards, Rockville, MD) in DMSO in 100 μL, followed by addition of an equal volume of buffer with NADPH (200 μM) and DTNB (6 mM) with reaction progress monitored at λ = 412 nm for TNB production. The concentration of DMSO in all reactions was 3.5%.

### Ethics statement

The *Loa loa* microfilariae donors were all adult male and female patients, aged 21 or older, residing in the Edea Health District of the Littoral Region of Cameroon. Ethical and administrative clearances were obtained from the Cameroon National Ethics Committee (N°2013/11/371/L/CNERSH/SP) and the Cameroon Ministry of Health, respectively. Written and signed informed consent was obtained from each participating patient, and all of them had 2000 *L. loa* microfilariae per mL of blood or greater. The patients were employed in the study as microfilariae donors only.

Animal studies were performed under the University of California San Francisco Institutional Animal Care and Use Committee (IACUC) approval #AN085723–02 and adhere to guidelines set forth in the NIH Guide for the Care and Use of Laboratory Animals and the USDA Animal Care Policies. Animals were euthanized by carbon dioxide inhalation followed by bilateral thoracotomy.

## Results

### Identification of auranofin as an effective drug that kills *Brugia* and *Onchocerca* worms *in vitro*

Results of the adult worm assay showed that the motility of female *B. pahangi* and *B. malayi* was inhibited by 97% within 18 hours of incubation with 3 μM of auranofin. Following our prescribed screening funnel, after this primary screen, auranofin was then assayed with adult female and male *O. ochengi*, *O. volvulus* L3, and *O. ochengi* and *L. loa* microfilariae.

Auranofin was highly effective in killing both male and female adult *Brugia* and *Onchocerca* worms and inhibiting molting of *O. volvulus* third-stage larvae to the fourth stage with IC_50_ values less than or equal to 1.1 μM (Table 1). Auranofin, however, was not very effective in killing *O. ochengi* and *L. loa* microfilariae. Auranofin’s IC_50_ value for adult female *O. ochengi* was 10 times lower than its IC_50_ value for *O. ochengi* microfilariae and 42.7 times lower than its IC_50_ value for *L. loa* microfilariae. This is an important consideration when treating individuals with auranofin in *L. loa* endemic areas.

**Table 1:** Effect of auranofin on filarial worms in vitro.

Species	Sex	Stage	Day	IC_50_ (uM)
*B. malayi*	Female	Adult	Day 3	1.1
	Male	Adult	Day 3	0.3
*B. pahangi*	Female	Adult	Day 3	0.5
	Male	Adult	Day 3	0.1
*O. ochengi*	Female	Adult	Day 7	0.3
	Male	Adult	Day 5	0.4
*O. volvulus*	-	L3	Day 6	0.3
*O. ochengi*	-	Microfilariae	Day 5	3.0
*L. loa*	-	Microfilariae	Day 5	12.8

### Structural features of auranofin-treated *B. pahangi* and *O. ochengi* adult worms

**Fig 1:**
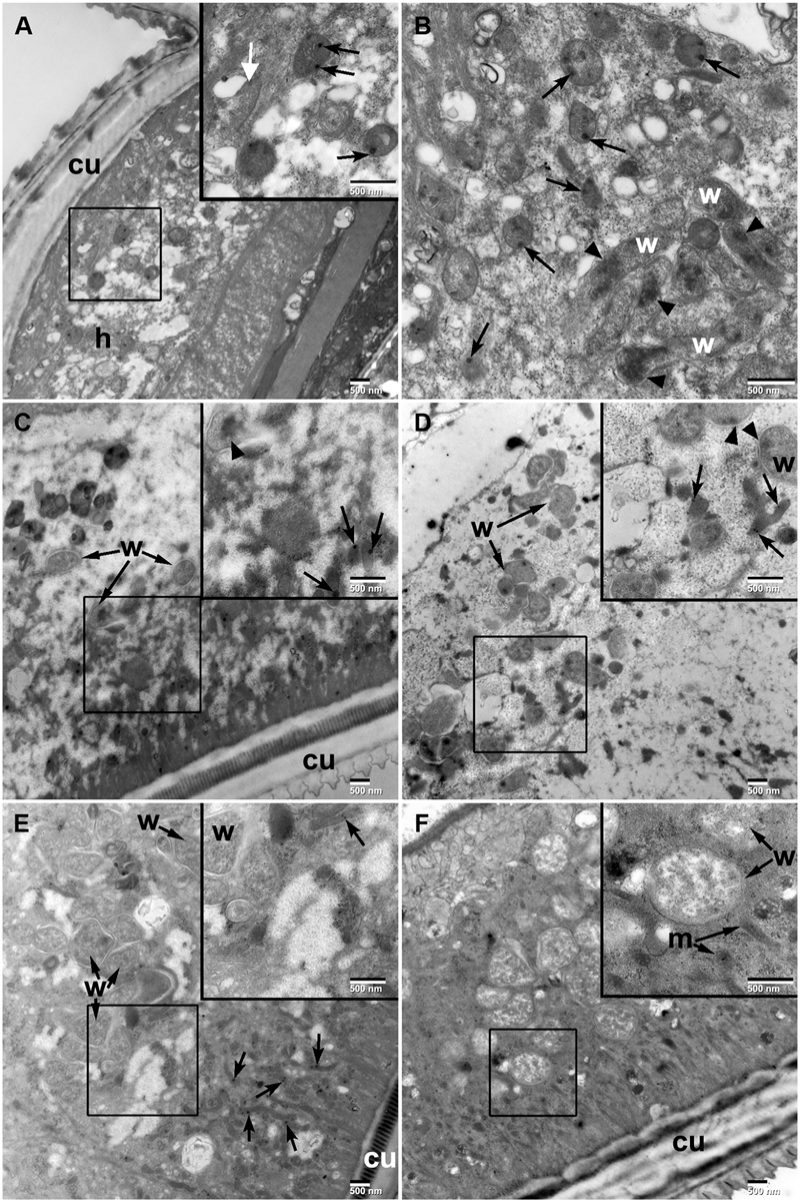
TEM images of auranofin treated *B. pahangi*. Transmission electron microscopy of auranofin treated versus control adult female *Brugia pahangi* after overnight drug treatment. (A) *B. pahangi* treated with 1 μM of auranofin. Hypodermal chord region (h) below cuticle (cu) of *B. pahangi* exhibiting vacuolation of tissue (compared to control worms, Fig. 1F). Insert; higher magnification of boxed region in (A) showing swollen mitochondria containing dark bodies (black arrows). White arrow indicates severely damaged mitochondrion. (B) *B. pahangi* treated with 1 μM of auranofin. High magnification of hypodermal chord region showing numerous swollen mitochondria containing dark bodies (black arrows) as well as shrunken *Wolbachia* (w) containing dark condensed material (black arrowheads) (compared to control worms, Fig. 1F). (C) *B. pahangi* treated with 0.3 μM of auranofin. Hypodermal chord region containing *Wolbachia* (black arrows) and dark bodies (white arrows). Insert; higher magnification of boxed region in (C) showing mitochondria containing dark bodies (black arrows) as well as *Wolbachia* (black arrowhead) containing condensed material. (D) *B. pahangi* treated with 0.1 μM of auranofin. Hypodermal chord region containing *Wolbachia* (black arrows). Inset; higher magnification of boxed region in (D) showing mitochondria containing dark bodies (black arrows) as well as *Wolbachia* (black arrowhead) containing condensed material. (E) *B. pahangi* treated with 10 μM of flubendazole. Hypodermal chord region containing *Wolbachia* (black arrows) and numerous mitochondria containing dark bodies (black concave arrows). Insert; higher magnification of boxed region in (E) showing a mitochondrion containing dark bodies (black arrows). (F) *B. pahangi* treated with 1% DMSO. Hypodermal chord region contains numerous *Wolbachia* without condensed material observed in auranofin treated cells. Insert; higher magnification of boxed region in (F) showing *Wolbachia* (w) as well as several mitochondria (m) without the dark bodies observed in treated cells.

Adult female *B. pahangi* incubated with 1 μM, 0.3 μM, or 0.1 μM auranofin overnight and adult female *O. ochengi* worms encapsulated in nodules incubated with 10 μM auranofin for 7 days were subjected to transmission electron microscopy to compare the internal morphology with their respective control female worms. Auranofin-treated *B. pahangi* worms showed considerable damage in the hypodermal region compared to control worms (Figs. 1a-1d). The hypodermal area of treated worms was highly vacuolated with remnants of swollen mitochondria containing dark bodies as well as shrunken *Wolbachia* containing dark condensed material. The hypodermal chord region of *B. pahangi* female worms treated with 10 μM of flubendazole contained normal *Wolbachia* with very few mitochondria containing dark bodies (Fig. 1e). In contrast, the hypodermal chord region in control worms (Fig. 1f) contained numerous *Wolbachia* without the condensed material observed in auranofin treated worms.

**Fig 2:**
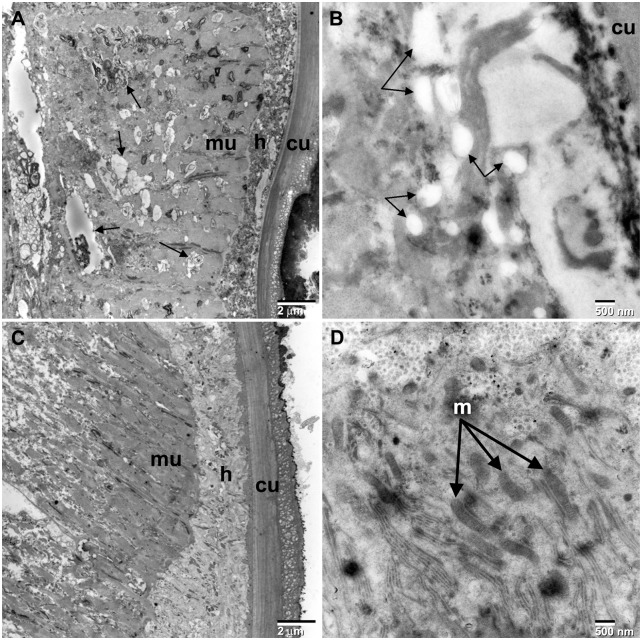
TEM images of auranofin treated *O. ochengi*. Transmission electron microscopy of auranofin treated versus control female *Onchocerca ochengi* 7 days post treatment. (A) Low magnification of *O. ochengi* treated with 10 μM auranofin. Numerous vacuoles with inclusion bodies (black arrows) were observed in the muscle tissue (mu) below the hypodermal chord (h). (B) High magnification of hypodermal chord region directly below the cuticle (cu). Numerous vacuoles (black arrows) were observed as was a complete absence of mitochondria. (C) Untreated *O. ochengi* exhibiting the typical arrangement of muscle (mu) and hypodermal chord (h) tissue below the cuticle (cu). (D) High magnification of hypodermal chord region directly below the cuticle showing numerous mitochondria (m).

Similar morphology was also observed in the *O. ochengi* auranofin treated worms (Fig. 2). Numerous vacuoles with inclusion bodies were observed in the muscle tissue below the hypodermal chord. Numerous vacuoles and a complete lack of mitochondria were also observed in the hypodermal chord region directly below the cuticle.

### Efficacy of auranofin on *Brugia* worms *in vivo*

Two *in vivo* studies were performed using the same dosing regimen of 5 mg/kg BID weekdays and SID weekends for 28 days (for a total of 48 doses). Study 1 and Study 2 are replicate studies, except that in Study 1 an interim necropsy was conducted to determine the plasma levels and level of infection 14 days after the first dose. The number of worms collected from these vehicle treated gerbils was 43 (13 male worms and 30 female worms, a ratio of approximately 1:2) and the total number of worms from the auranofin treated gerbils was 11 (4 males and 7 females, a ratio of approximately 1:2).

**Fig 3:**
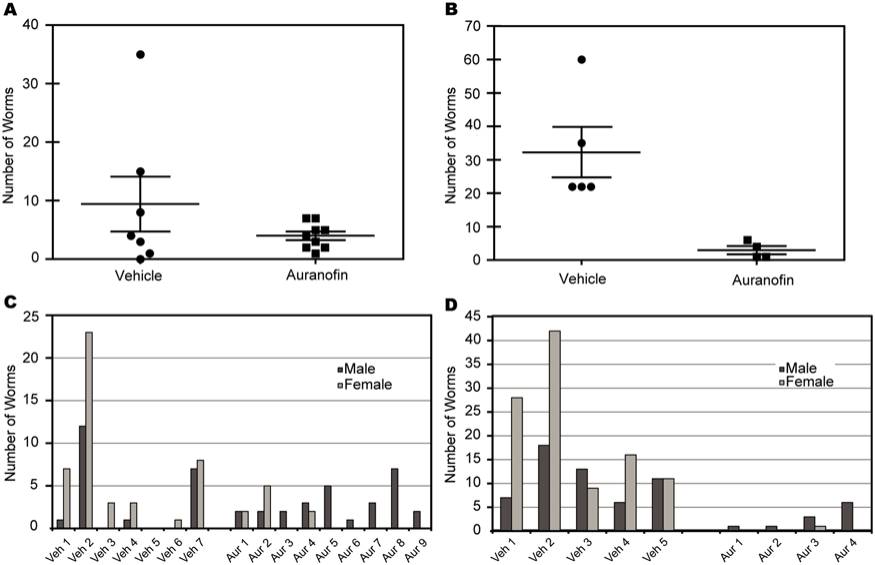
Worm retrieval from *B. pahangi* infected gerbils treated with auranofin. Total worms recovered from (A) Study 1 and (B) Study 2 of gerbils treated with 5 mg/kg auranofin or vehicle with 48 doses for 28 days. Fig. 3A and 3C also include worms recovered from interim necropsy gerbils treated for 14 days. The difference in total worm retrieval between auranofin treated and vehicle treated gerbils in Study 2 was statistically significant (p < 0.05). Male and female worms recovered from (C) Study 1 and (D) Study 2.

In Study 1, the average number of worms from all vehicle treated animals (n = 7) was 9.4 worms and the average number of worms from all treated animals (n = 9) was 4.0 worms (Fig. 3A). There was a 58% overall reduction in worm burden in the auranofin treated group in comparison with the vehicle treated group but difference between the two groups was not statistically significant (p > 0.05). In the control group the ratio of male to female worms at terminal necropsy was 1:2, similar to the ratios found in the control group and treated group at the interim necropsy. In the treated group however, the ratio of male to female worms was 12:1 at terminal necropsy. This sex ratio bias was also observed in the auranofin treated group in Study 2 (Fig. 3B).

In Study 2, there was a 91% reduction in worm burden in the auranofin treated group compared to the control group, which was statistically significant (p = 0.01) in a Student’s T-Test. There were 161 total worms recovered from the vehicle group (mean = 32 worms per gerbil), of which 55 were males and 106 were females (ratio of 1:2). In the auranofin treated group, there were a total of 12 worms recovered (mean = 3 worms per gerbil): 11 were males and only 1 was a female worm (ratio of 11:1) (Fig. 3D). This remaining female was encapsulated with host tissue.

Plasma collected from the necropsies from Study 1 and Study 2 was submitted for elemental gold analysis (Table 2). Gold was not detected in the vehicle group. Plasma taken 2 hours after gerbils were given an auranofin dose (but had been treated for 14 days) had gold levels of 5.08 μM and 8.63 μM. In Study 1 and Study 2, the mean plasma gold levels 16 days after the last dose were 701 nM and 609 nM, respectively. There were 2 animals in each of the treatment groups that did not have detectable levels of gold in their plasma but this may be due to the limit of detection in the assay, where any value less than 100 μg/L (508 nM) gold is given as zero.

**Table 2:** Plasma gold levels from Brugia infected gerbils following necropsy.

		Time after last dose	Plasma gold levels (ug/L)	Concentration of gold (uM)
**Study #1**	Aur1	2 hours*	1700	8.631
	Aur2	2 hours*	1000	5.077
	Aur3	11 days	270	1.371
	Aur4	14 days	340	1.726
	Aur5	16 days	160	0.812
	Aur6	16 days	170	0.863
	Aur7	16 days	0	0
	Aur8	16 days	170	0.863
	Aur9	16 days	190	0.965
**Study #2**	Aur1	16 days	130	0.660
	Aur2	16 days	190	0.965
	Aur3	16 days	0	0
	Aur4	16 days	160	0.812

* Plasma taken at interim necropsy, two hours after last dose (following 14 days of treatment).

### TrxR activity decreased in adult female *Brugia* after treatment with auranofin *in vitro* and *in vivo*

**Fig 4:**
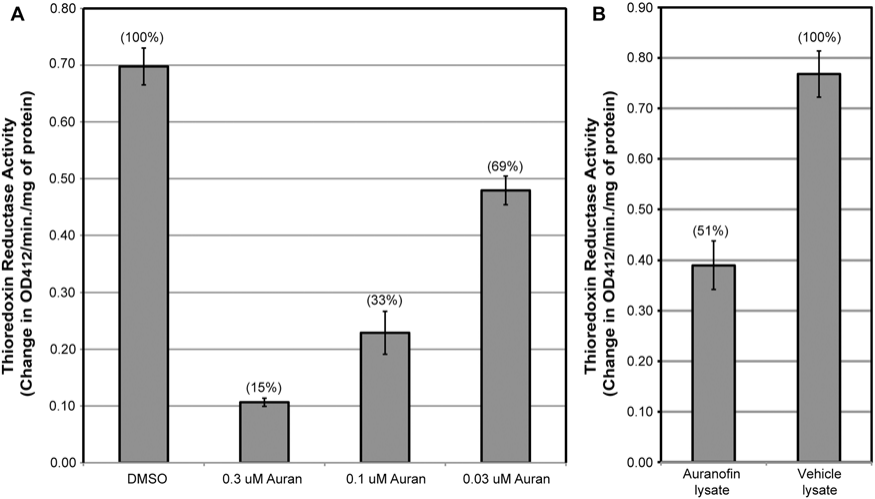
Thioredoxin reductase activity in auranofin treated Brugia spp. (A) Activity of endogenous *Brugia* thioredoxin reductase from soluble worm lysates following incubation with 1% DMSO or 0.3 μM, 0.1 μM, or 0.03 μM of auranofin *in vitro*. Percentages indicate the percent activity of TrxR compared to DMSO controls. (B) Enzymatic activity of worms collected 16 days after the last dose from gerbils treated with auranofin or vehicle. The lysate of worms taken from gerbils treated with auranofin shows 49% less thioredoxin reductase activity than those taken from gerbils treated with vehicle only. Percentages indicate the percent activity of TrxR compared to vehicle controls.

Thioredoxin reductase activity in *Brugia* females cultured for 5 hours with 0.3 μM, 0.1 μM or 0.03 μM of auranofin *in vitro* was significantly reduced (p < 0.05) to 15%, 33% and 69% of endogenous activity, respectively, compared to the activity in DMSO-treated worms (Fig. 4A).

When *Brugia* worms were removed 16 days after the last dose from gerbils treated with auranofin *in vivo*, endogenous enzyme activity was reduced significantly (p < 0.05) by 49% compared to worms collected from vehicle treated gerbils (Fig. 4B). These data further suggest that endogenous *Brugia* TrxR is specifically inhibited by auranofin.

### Activity of recombinant *B. malayi* TrxR is inhibited by auranofin

Recombinant *B. malayi* TrxR (rBmTrxR) was overexpressed in *E*. *coli* at approximately 10 mg of protein per liter of culture following His-Trap affinity chromatography. Two organic gold compounds, auranofin and aurothioglucose, were assayed with rBmTrxR and both were found to be effective inhibitors suggesting that gold is the active component of auranofin as expected from previous studies with TrxR and thioredoxin glutathione reductase [24,25]. Both compounds had inhibitory activity in the low nanomolar range with auranofin IC_50_ = 3 nM and aurothioglucose IC_50_ = 9 nM. Production of eukaryotic Sec-proteins in bacteria is not 100% efficient. The misreading of the Sec codon (UGA) results in premature termination of the peptide resulting in an enzymatically inactive product [29]. Since the active and inactive proteins both bind metal affinity resins and differ in size by only two amino acids, recombinant protein is a mixture of both active and inactive enzyme forms. Based on previous studies [29–31] between 10% and 20% of the protein is active, with the remainder inactive. The inhibitory activity of both compounds indicates that they irreversibly inhibit rBmTrxR at a one-to-one molar ratio, with potencies similar to those found for other TrxR and thioredoxin glutathione reductase enzymes [32,33].

## Discussion

The main goal of our study was to identify macrofilaricidal drugs for the treatment of onchocerciasis and LF. Two major challenges in developing new drugs for these neglected diseases are finding suitable animal models for preclinical studies and limiting the costs of drug development and production. To date, the only animals in which *O. volvulus* can develop to patency are chimpanzees and mangabey monkeys [34–36]. *O. ochengi*, which infects cows, is thought to be closely related to *O. volvulus* [37], and previous studies have used *O. ochengi* as a model for *O. volvulus* infection [13–15]. *Brugia malayi* and *B. pahangi*, as members of the Filariidae family, are also closely related to *O. volvulus* [38]. Because of the large number of compounds required to identify preclinical candidates and with the accessibility of large numbers of adult worms that can be collected from gerbils, we selected adult *Brugia* for our primary screens. Following our funneling scheme, we first identify compounds screened with adult female *Brugia* in the Worminator assays. Compounds that inhibit motility by 75% compared with control worms are then screened against *O. volvulus* molting larvae and *O. ochengi* adult worms in an MTT assay and motility assay.

In an effort to identify candidate drugs that could be more rapidly moved into clinical trials, we screened an FDA-approved library of compounds and found that auranofin was effective in killing adult *Brugia* and *O. ochengi* worms and in inhibiting larval *O. volvulus* from molting from L3s to L4s *in vitro*. Microfilariae of *O. ochengi* and *L. loa* were used in a counter screen to determine the effects of auranofin on the microfilarial stage. We found that the IC_50_s for *O. ochengi* and *L. loa* microfilariae were approximately 10 and 42.7 times higher, respectively, compared with the IC_50_s of adult female *O. ochengi*. These results may have important implications, should auranofin be used for treatment in areas endemic for both onchocerciasis and loaiasis to avoid severe adverse events.

Auranofin was then tested for its efficacy in secondary screens with infected gerbils. Results of the *in vivo* studies showed that dosing animals for 28 days at 5 mg/kg was effective in reducing worm burden by 58% and 91% in the two studies. Gold plasma levels in gerbils obtained at 2 hours post-dose after 2 weeks of treatment indicated that the plasma gold levels were in the micromolar range (5.08 μM and 8.63 μM), approximately 5 to 10-fold higher than the IC_50_s from the *in vitro* worm assays. These gerbils continued to maintain gold levels in their blood approximately 2 weeks after the last dose (0.66 μM) which may suggest that a sustained level of gold is necessary for worm killing.

Transmission electron micrographs of adult *Brugia* incubated overnight with 1 μM auranofin showed that there was extensive damage to *Wolbachia* in the hypodermal area, in contrast to worms treated with 10 μM flubendazole. Flubendazole at this concentration did not cause vacuolization but only minor changes to the mitochondria, which appeared to contain black bodies. Loss of integrity in muscle tissue and the hypodermal chord were also observed when *O. ochengi* adults were incubated with auranofin at 10 μM for 7 days. Thus, the structural damage caused by auranofin is similar in both species, except that presumably due to the large size of *O. ochengi*, auranofin takes a much longer time and higher concentrations of drug to have an effect.

Auranofin is an FDA-approved drug that was originally developed to treat rheumatoid arthritis. There is strong evidence in several species of parasites that thioredoxin reductase and a similar enzyme, thioredoxin glutathione reductase (TGR), are targeted by auranofin [26,33,39–41]. Previous studies have shown that this drug is an effective antiparasitic agent against a number of organisms, including *Schistosoma mansoni* and *S*. *japonicum* [33,42], *Echinococcus granulosus* [43], *Taenia crassiceps* [44], *Plasmodium falciparum* [45], *Leishmania* spp. [46], *Trypanosoma brucei* [47], *Giardia lamblia* [39,48] and *Toxoplasma gondii* [49]. In animal studies, auranofin was highly efficacious in treating amoebic colitis in mice and amoebic liver abscesses in hamsters [26]. Auranofin treatment also significantly decreased worm burdens in mice infected with *S*. *mansoni* [33] and suppressed footpad lesion formation and reduced existing lesions in a mouse model of cutaneous leishmaniasis [46].

The thioredoxin system is integral to maintaining a reduced state and managing oxidative stress within the cell, which makes this system critical for organism survival [50]. Thioredoxin, which is reduced by thioredoxin reductase, is a substrate for redox enzymes including peroxidases in filarial worms [18,51]. Inhibition of TrxR by auranofin alters the redox state of the cell leading to an increased production of hydrogen peroxide and oxidation of the components of the thioredoxin system thereby enhancing apoptosis [52]. Sayed et al (2006) found that silencing peroxiredoxins, downstream redox partners of TrxR, in schistosomes led to detectable protein and lipid oxidation [53]. Inhibition of *Brugia* TrxR by auranofin may disrupt this process in filarial worms, which can then lead to worm death. Interestingly, there were significantly fewer female worms than male worms from gerbils treated with auranofin. The preferential killing of female worms may be due to the host’s immune response against females when they release microfilariae [54,55]. It is also possible that as female worms develop and molt from the larval stage to the adult stage, they elicit an immune response that, together with auranofin, preferentially kills female worms over males.

The mode of action of auranofin is thought to be a specific inhibition of the selenoenzymes thioredoxin reductase (TrxR) and thioredoxin glutathione reductase (TGR). No TGRs from *Brugia* have been identified thus far. Kuntz et al (2007) showed that auranofin inhibited TGR in adult schistosomes *in vitro* but had no effect on the activities of another selenoenzyme, glutathione peroxidase, or the abundant enzyme lactate dehydrogenase [33]. Loss of TGR activity preceded parasite death, indicating that specific inhibition of TGR by auranofin was responsible for parasite death in schistosomes.

Auranofin inhibition has also been shown to be less specific to glutathione peroxidase and glutathione reductase, which have about 1000-fold higher IC_50_s compared to TrxR isolated from human placenta [32]. Other thioenzymes, such as the cysteine protease cathepsin B, also had significantly higher IC_50_s when tested with auranofin (approximately 250 μM) [56] compared with the IC_50_ of auranofin with rBmTrxR.

Thioredoxin reductase enzyme activity of *B. malayi* adult worms treated with auranofin was significantly lower compared to with vehicle-treated worms in the *in vitro* assays. TrxR activity was also decreased by 49% in worms removed from gerbils 16 days after treatment with auranofin, supporting the hypothesis that auranofin specifically targets TrxR in these worms.

Targeting the thioredoxin system by inhibiting thioredoxin reductase may be a promising strategy for treating filarial infections, since the enzyme appears to be necessary for worm survival. It is possible that auranofin treatment increases the susceptibility of the parasite to oxidative damage, which in turn allows the host’s immune system to eliminate the parasite.

Since auranofin is already an FDA-approved drug, the path to clinical trials is streamlined. Patients with rheumatoid arthritis who were treated with auranofin for an average of 6 months had few side effects, with the most common side effect being diarrhea [20]. In the present study auranofin was shown to be efficacious in the *Brugia*/gerbil model when given for 28 days. Additional studies will be conducted to determine efficacy with shorter treatment regimens and to obtain pharmacokinetic data. Auranofin will also be evaluated for any synergistic effects with other drugs such as doxycycline and for its use as part of a macrofilaricidal cocktail.

## Supporting Information

S1 Text This file contains detailed information on the process used to verify that *B. malayi* TrxR is a selenoprotein. (DOC)
