# Supplementary material for: Repurposing Auranofin as a Lead Candidate for Treatment of Lymphatic Filariasis and Onchocerciasis
Source: PLoS Negl Trop Dis. 2015 Feb 20;9(2):e0003534. doi: 10.1371/journal.pntd.0003534 (PMC4336141; doi:10.1371/journal.pntd.0003534)
Supplement: S1 Text — (DOC) [file pntd.0003534.s001.doc]

**S1 Text**

To determine if the *B. malayi* TrxR is a selenoprotein, as are some known thioredoxin reductases, the *B. malayi* genome was searched for sequences similar to both the published *Caenorhabditis elegans* SECIS [1] and a highly similar sequence in *O. volvulus* thought to be a SECIS. First, the *O. volvulus* genome assembly was searched for the published *C. elegans* SECIS via the Wellcome Trust Sanger Institute BLAST server, which yielded a sequence with 83% identity.

*C. elegans* SECIS:

5’-GTGACGACCTTTGGCTAAACTCCATCGTGAGCGCCTCTGGTCTGATG-3’

*O. volvulus* SECIS:

5’-GTGACGAATTTGAGCTAAACTCCATTGTGAGCGCTCATTTTCTGATG-3’

The full *O. volvulus* genome was searched for the *O. volvulus* SECIS, and the nucleotide sequence from 300 bases upstream of the start of the SECIS through 100 bases downstream from the end of the SECIS were translated in EXPASY. One of the resulting reading frames included amino acids 617 through 636 of the *O. volvulus* TrxR sequence followed by a stop codon (TGA), glycine, then another stop codon (TAA). Given the proximity to the SECIS, that selenocysteine is encoded by a UGA codon, and that other Sec-containing TrxR have the C-terminal amino acid motif Gly-Cys-Sec-Gly, the TGA is most likely a selenocysteine followed by a glycine.

Once the *O. volvulus* SECIS was located, the amino acid sequence of *B*. *malayi* TrxR (XP_001898729.1) was found in the NCBI database. This sequence also ended with a glycine followed by a cysteine and then a TGA termination codon. To see if this sequence was mistakenly truncated, a BLAST search was used to compare the *O. volvulus* SECIS with the *B. malayi* whole genome shotgun sequence (NW_001893021.1). This yielded a matched reverse complement sequence in the *B. malayi* genome.

*B. malayi* SECIS (reverse complement of sequence found in genome as it appears in NCBI):

5’- GTGACGAATTTGGACTAAACTCCATCGTGAGCGTTCATTTTCTGAT-3’

The reverse complement of the last 50 nucleotides of the *B. malayi* TrxR partial mRNA sequence (XM_001898694.1) was generated through <http://reverse-complement.com>. The reverse complement section of the TrxR mRNA and the *B. malayi* SECIS were found in the *B. malayi* genome separated by 122 amino acids. The SECIS can be found as far as 5 kb from the selenocysteine codon [2]; therefore, this SECIS likely corresponds to the *B. malayi* thioredoxin reductase. When this partial sequence of the *B. malayi* genome was translated through EXPASY, the resulting amino acid sequence ended with Gly-Cys-stop-Gly-stop; as with the *O. volvulus* sequence, this second to last stop is probably a selenocysteine followed by a glycine.

1. Buettner C, Harney JW, Berry MJ (1999) The *Caenorhabditis elegans* Homologue of Thioredoxin Reductase Contains a Selenocysteine Insertion Sequence (SECIS) Element That Differs from Mammalian SECIS Elements but Directs Selenocysteine Incorporation. Journal of Biological Chemistry 274: 21598-21602.

2. Fagegaltier D, Lescure A, Walczak R, Carbon P, Krol A (2000) Structural analysis of new local features in SECIS RNA hairpins. Nucleic Acids Res 28: 2679-2689.
